# Sensors for Structural Health Monitoring of Agricultural Structures

**DOI:** 10.3390/s21010314

**Published:** 2021-01-05

**Authors:** Chrysanthos Maraveas, Thomas Bartzanas

**Affiliations:** 1 Department of Civil Engineering, University of Patras, 26500 Patra, Greece; 2 Department of Natural Resources and Agricultural Engineering, Agricultural University of Athens, 11855 Athens, Greece; t.bartzanas@aua.gr

**Keywords:** sensors, agricultural structures, health diagnosis

## Abstract

The health diagnosis of agricultural structures is critical to detecting damages such as cracks in concrete, corrosion, spalling, and delamination. Agricultural structures are susceptible to environmental degradation due to frequent exposure to water, organic effluent, farm chemicals, structural loading, and unloading. Various sensors have been employed for accurate and real-time monitoring of agricultural building structures, including electrochemical, ultrasonic, fiber-optic, piezoelectric, wireless, fiber Bragg grating sensors, and self-sensing concrete. The cost–benefits of each type of sensor and utility in a farm environment are explored in the review. Current literature suggests that the functionality of sensors has improved with progress in technology. Notable improvements made with the progress in technology include better accuracy of the measurements, reduction of signal-to-noise ratio, and transmission speed, and the deployment of machine learning, deep learning, and artificial intelligence in smart IoT-based agriculture. Key challenges include inconsistent installation of sensors in farm structures, technical constraints, and lack of support infrastructure, awareness, and preference for traditional inspection methods.

## 1. Introduction

The review article focuses on utilizing sensors in agricultural structures to accurately monitor the durability of concrete, metallic structures, and greenhouse covering materials and susceptibility to environmental degradation. For example, wood structures in farms are easily degraded by water [1] while UV radiation negatively affects polymer materials used in the construction of greenhouses. The scope of the review focuses on sensors for measuring chloride ingress [2], corrosion activity, carbonation, concrete (electrical) resistivity, permittivity, and stress/strain [3] and the following thematic areas: sensors for monitoring and health diagnosis of agricultural structures, sensors for concrete, steel, timber [4], electrochemical, fiber optic, wireless, ultrasonic, fiber Bragg grating sensors, self-sensing materials, the utility of sensors in facilitating the repair process, mitigation of failure, initiation of appropriate action, improving the durability and useful life, and the replacement of agricultural structures. The measurement of corrosion activity is critical in steel farm structures, given the higher susceptibility to redox reactions and gradual environmental degradation [5,6].

The focus on agricultural structures (silos, greenhouses, storage areas, and general housing) is justified by the cost implications of material failure/structural integrity loss. For example, the construction of a greenhouse structure costs about $65–85 per square meter [7]; the costs could be higher depending on the type of greenhouse and the fact that structural damage would have a direct negative impact on the return (NR) from greenhouse production. The structural damage of concrete, steel, and greenhouse structures requires extensive repairs, which are resource-intensive and time-consuming. Recent studies estimate that the cost of repairing an agricultural silo varies between $10,000 and $131,250 [8]. Considering that such costs are unsustainable, the adoption of preventative measures such as installing sensors is necessary.

Beyond grain silos, the repair of individual farm structures after structural failure is costly and ineffective, considering the size factor, cost of construction, function, age, and material modification of the building structures. Beyond the farm environment, the state of the art review of sensors has practical implications for the global construction and real estate sectors, which have recorded significant losses from the failure or continued application of functionally obsolete building structures. For example, at least one in four US bridges are functionally obsolete and at a higher risk of failure [9]. The total cost of repairing functionally obsolete structures in the US is about $3.6 trillion [9]. The costs could be higher because there are no recent estimates. The risk of structural failure was not unique to bridges considering that different structural failure forms were observed in building structures in Australia’s New South Wales [10]. Similar challenges were documented in developing economies such as India and Iran [11], where non-adherence to building codes is common.

Considering that it is impractical for developers and real estate investors, and individual owners to raise trillions of dollars to repair buildings, the monitoring of agricultural structures is critical to commercial agricultural production, especially in the West, where harsh weathers limit the agricultural production cycles and necessitate the installation of greenhouse structures [7,12]. Failure analyses of structural damage of silo structures have established that corrosion of steel reinforcement, water, chemical attack, absence of horizontal and vertical reinforcements [13], and non-compliance or failure of the building design codes leads to structural damage. The commonly applied building codes for silos include AS 3774-1996, ANSI/ASAE EP433 DEC1988, ACI 313-97, and BS EN 1991-4:2006 [6]. Secondary causes of failure include wall damage, connection failures, and the lack of adequate beam support [14]. Such risks can be mitigated through real-time monitoring, which provides a realistic assessment and prediction of the structure’s in-service performance. According to Lynskey [14], there are visible warning signs before structural failure, including concrete spalling and delamination, consistent cracking patterns, the formation of concrete chunks. However, such signs of potential failure might be located in the interior or top sections of a structure. In such cases, it is difficult to inspect the damage. Such challenges can be mitigated using sensors. Apart from mitigating the risk of sudden structural failure, installing sensors for monitoring could provide owners with actionable information that facilitates future planning.

**Figure 1:**
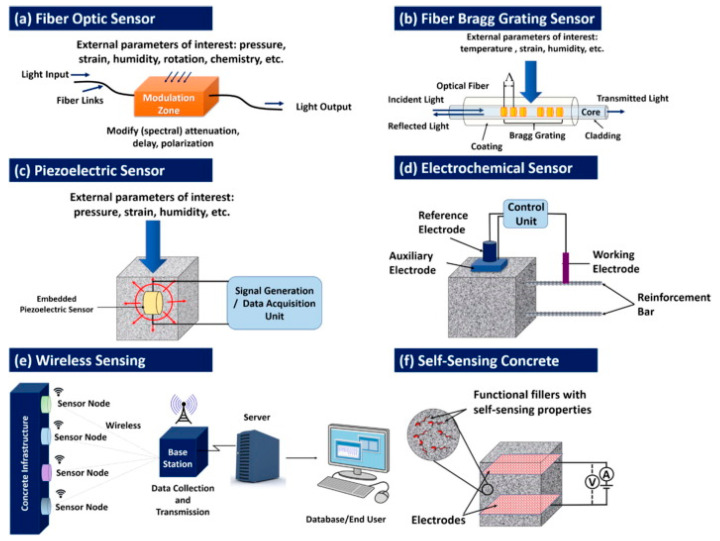
Types of sensors for structural health monitoring [15].

The sensors for health diagnosis of farm structures and buildings are grouped into electrochemical sensors, fiber-optic sensors, piezoelectric sensors, wireless sensors, fiber Bragg grating sensors, and self-sensing concrete [15] as shown in Figure 1. Each type of sensor serves a unique function in the monitoring process. For example, the fiber Bragg grating sensors undertake multiple and simultaneous measurements across the optical fiber’s entire length [9]. Such properties contribute to the suitability of the sensors in steel and reinforced concrete structures. However, the selection of one type of sensor over another is based on multiple technical criteria, including bandwidth, resolution, type of the transducer, accuracy, signal to noise ratio, options for fiber Bragg grating, Raman scattering and SOFO, [16] and previous application of the technology in monitoring agricultural buildings.

The classification scheme presented in Figure 1 excludes non-structural sensors, such as gas sensors, which monitor NH_3_ and H_2_S levels in farms and other environments [17]. Accumulation of NH_3_ gas in the atmosphere is a common phenomenon on farms due to the application of nitrogen-containing fertilizers [18]. High levels of ammonia in the environment are characterized by particulate matter exceeding PM 2.5. The accumulation of ammonia particulate matter could have a deleterious impact on concrete sustainability because of the following reasons. The diffusion of ammonia gas across the concrete structure increases the porosity and permeability of the wall [19]. High levels of concrete permeability have been correlated with a pronounced risk of structural failure due to water seepage and chemical attack [20]. However, the risk of material permeability could be mitigated by material modification. Experimental studies have demonstrated that silica fumes suppress permeability to chloride and water intrusion and consequently improve the structure’s durability [21]. However, material modification might not be a practical alternative for new and old farm structures constructed with standard materials. In brief, even though the discussion’s scope is limited to structural health monitoring, gas sensors contribute to the durability of building structures, especially in farms where nitrogen fertilizers are applied. In theory, gas sensors are appropriate based on cost and technical considerations—there are fewer technical requirements for intelligent agriculture. However, there is limited evidence of practical application in commercial farms.

### 1.1. Benefits of Deploying Sensors in Structural Health Monitoring

Sensors for structural health monitoring offer distinct advantages compared to other methods used to monitor the health of concrete structures including, controlled monitoring of the risk of corrosion without compromising the structural integrity of the building (non-destructive testing—NDT) [22,23,24]. The NDT process involves the extraction of a tiny sample from the building structure. Secondary benefits include the ability to monitor large sensing areas under harsh environments (mechanical strength, moisture, and fill materials), long-term [25], in situ, and real-time monitoring [26]. However, these benefits are not universal—they are sensor specific. For example, the cost benefits associated with piezoelectric sensors are offset by the gradual degradation of the transducer’s mechanical/electrical properties [27]. Similarly, the accurate assessment of concrete damage using electrochemical sensors is compromised by challenges in image reconstruction techniques [28]. Based on these considerations, the selection process should be informed by the sensors’ accuracy and reliability rather than cost or size.

The non-destructive nature of sensors is critical because traditional visual and technical inspection methods were often intrusive and interrupted the structure’s normal functioning [29]. Additionally, Hoult et al. [9] note that visual inspection methods cannot accurately determine the extent of fatigue leading to cracking and the mass losses due to corrosion. In contrast, microscopic samples extracted using sensors enable engineers to examine the state of the building materials in detail, correlate the sensor signal depending on the intensity of corrosion, and predict various what-if scenarios, such as the impact of cathodic protection systems, interruption of the cathodic protection systems, stress levels, heat treatment, and material surface finish [30]. In other cases, sensors offer an accurate diagnosis of the rate of corrosion. The monitoring of the structures is critical given the high risk of exposure to mechanical loading (especially in grain silos), water and moisture, and agricultural chemicals, which trigger chemical attacks [31,32,33,34]. Periodical exposure to chemical exacerbates the risk of structural damage and failure of farm structures. The functionality, accuracy, and reliability of each sensor type in diagnosing the general health of agricultural building structures and smart and intelligent systems under development are discussed to provide better insights on future technologies, opportunities, and challenges.

A comparative analysis of the costs of different sensors is presented in Table 1. The data confirms that all types of sensors for structural health monitoring are expensive, except for the single-mode bare fiber [35]. However, from a technical point of view, the cost might not be a primary consideration given the role of sensitivity, reversibility, response time, and the sensing principle on the measurements’ accuracy.

The durability, operability, and survivability of sensor components are critical considerations that should be taken into account during the analysis process. The risk of sensor failure or damage abounds regardless of whether the sensor is embedded into the building structure or bonded on the surface of the targeted building structure [36]. The functionality of the electrical components degrade over time; this, in turn, impacts the accuracy of the measurements [37]. Smart and intelligent systems can partly mitigate the constraints in sensors.

Apart from the costs and technical requirements, the ecological effects should be taken into consideration. The chemical composition of electrochemical sensors could harm human health if the contents leak into farm structures such as silos where edible grains are stored. Despite the drawbacks of sensor-based technologies, manual inspections are unfeasible given they are labor and resource-intensive; this means that the selection of appropriate sensor technology should be informed by a wide array of factors, especially considering that the mitigation of structural damage is incomparable to rebuilding. 

### 1.2. Smart and Intelligent Systems

New sensor technologies integrate machine learning, deep learning [38], and artificial intelligence [39,40] for structural health monitoring and sensor fault detection [41]. Apart from structural health monitoring, the sensors can support a wide array of applications, including smart farming, by monitoring the quantity and quality of stock in silos [42]. In other cases, sensors are integrated into vibration, acoustic, pressure-based, radar, in situ, imagery, camera, and laser technologies for structural health monitoring [38]. Azimi et al. [40] reported that convolutional neural network (CNN)-based approaches and models enabled engineers to identify damage and localize repairs; this was largely achieved through transfer learning techniques. Similarly, Yuan et al. [39] acknowledged that the integration of machine learning systems was essential. However, in place of the CNN-based methods, the latter study focused on Multi-layer feed-forward artificial neural networks (ANNs), which were proven to be among the best machine learning methods for automated damage detection.

On the downside, the widespread application of smart technologies was compromised by the absence of data that accurately represents the different damage scenarios; this is particularly true for farm settings. In general, the accuracy of machine learning and artificial intelligence-based sensors is largely dependent on the availability of accurate data. Similar observations were made by Serov [43], who argued that automatic construction of hierarchical description of the physical world was a fundamental challenge facing artificial intelligence-based structural health monitoring systems [43]. Such limitations illustrate that smart technologies cannot entirely replace human consciousness; this means that human inspections might be necessary to verify the smart and intelligent assessments of building structures’ structural health. Other concerns include the limited capacity of conventional systems to process large volumes of data.

From a long-term perspective, there are tangible cost benefits that could accrue from the adoption of smart and intelligent technologies. The existing challenges could be partly resolved by integrating domain knowledge and refinement of the existing machine learning and deep learning technologies. Alternatively, dynamic artificial neural network (DANN) could help mitigate the data constraints by learning linear and non-linear input data streams [43]. Given the preliminary outcomes, the adoption of smart and intelligent systems involves a tradeoff between cost [38], accuracy, and uncertainty. A fundamental question is whether advances in technologies would facilitate the widespread adoption of machine learning and AI-based sensors in farm environments—present research evidence is inconclusive. However, two scenarios were possible.

On the one hand, technological progress might facilitate the development of ANN, CNN, and alternative sensors that are reliable in structural monitoring. The scenario is largely possible considering that significant advances had been made in developing smart home solutions that integrate various types of sensors, including IoT [44], smart plug meters, air conditioning, temperature monitoring devices, and activity monitoring. The researcher postulates that the insights drawn from association rules mining, autoregressive integrated moving average, reinforcement learning, and dimensionality reduction [45] in machine learning and artificial intelligence applications for smart homes could be applied to structural health monitoring. The need for technical expertise is demonstrated by the complexities of the fault detection and isolation (FDI)-based structural health monitoring algorithms [41]. On the other hand, widespread application in farms could be impeded by cost consideration and the absence of technical expertise to interpret the data.

The second scenario would entail the maintenance of the status quo. Owners of farm structures would continue to rely on electrochemical sensors, fiber Bragg grating sensors, self-sensing concrete [15], fiber-optic sensors, piezoelectric sensors, and wireless sensors that are based on non-AI and non-machine learning methods; this is possible considering there are limited accounts of cases in which AI and machine learning solutions have been successfully deployed in farm-based structural sensors. Nonetheless, the potential application has been demonstrated using a simulation of an offshore wind farm using virtual SCADA data [46]. Considering that the simulations were successful, the intelligent sensors could be useful in remote farm environments. The cost benefits of sensors for structural monitoring are discussed in the next section taking into account signal transmission, signal to noise ratio, and other technical limitations.

### 1.3. Cost–Benefits of Sensors for Structural Health Monitoring

From a theoretical point of view, there are multiple benefits associated with installing sensors, including a reduction in the maintenance and operation costs, durability, and functionality of the building structure. Early detection of structural damage could save human and animal lives [47] in residential buildings and commercial agriculture, respectively. Historical evidence indicates that catastrophic damage induced by earthquakes, tsunamis, and other natural phenomena often results in many fatalities; this is especially true in developing countries where adherence to construction standards remains low [11]. The impact of construction standards on building collapse is supported by statistical data from the UK, US, Asia, and the Middle East [11,48]. Earthquakes of similar magnitudes were reported in California, the US, and Bam, Iran, but 41,000 deaths were reported in the latter and only two in the former [11]. The non-adherence to building codes and zoning was cited as a critical challenge in the latter. The non-adherence to building codes is detrimental to commercial agriculture, especially poultry production in concrete buildings and wooden structures which are susceptible to water-induced fungal attacks and degradation [49,50].

Considering that adherence to building standards would require a cultural shift in construction practices and radical changes in the political economy [11], the real-time monitoring of buildings using sensors provides a reprieve for residential and commercial building owners and inhabitants. However, the utility of sensors is impacted by the critical technical and economic constraints that impede the widespread adoption of sensors; this means that despite the clear evidence in support of the installation of sensors to mitigate material failure in agricultural systems, there are divergent views on the need to install sensors in buildings. For example, installing sensors can be contested in regions with a large stock of historical building structures [51,52,53]. In such cases, strict adherence to building standards does not offset the risk of structural failure.

The risk of failure in historical building structures is exacerbated by continuous exposure to the elements. One study estimated that the cost of restoring structurally deficient or functionally obsolete buildings in the US is $3.5 trillion [9]. Since it is not practical to spend such resources on structural repairs, real-time monitoring was indispensable to forestall catastrophic damage.

One school of thought supports the installation of sensors for real-time monitoring. The school of thought is grounded on the innumerable benefits associated with modern sensors. Another school of thought does not support installing sensors because of economic considerations, durability, survivability, and other technical considerations such as the overall low probability of catastrophic failure for well-designed building structures. The latter point of view is reinforced by Taheri’s [15] research, which confirmed that reliable “structural health monitoring relies on the employment of multiple sensors in different locations of a structure, which can be quite costly” [15]. Similarly, Hoult et al. [9] noted that the installation of strain gauges/conventional sensors might not offer a representative picture of structural defects because the damage is localized in specific non-pre-defined areas. The positioning of the agricultural building sensors might yield inaccurate data primarily because structural deformation is often not localized in one area/predefined location [9]. A priori knowledge of the agricultural structure is necessary for accurate prediction of areas with a high risk of material failure.

The observations made by Taheri [15] are not representative, given that there are affordable sensors for structural health monitoring. Kintner-Meyer et al. [37] note that the cost of sensors for monitoring the structural health of building structures can be mitigated through the selective installation of sensors. In particular, the study identified wireless sensors as cost-effective—the elimination of connection wires translates to 20% savings. Similarly, fiber Bragg’s sensors are inexpensive [15]. On the downside, the cost savings involve a tradeoff with the signal distance, which varies between 30 ft to 2500 ft in an open field or 15–35 miles within the line of sight [37]; this is demonstrated in Table 2. Beyond the cost savings, the wireless sensors impact the signal-to-noise ratios and transmission speeds [15]; this, in turn, has a domino impact on the strength and accuracy of the measurements. Secondary concerns relate to high energy consumption.

### 1.4. PRISMA Guidelines

**Figure 2:**
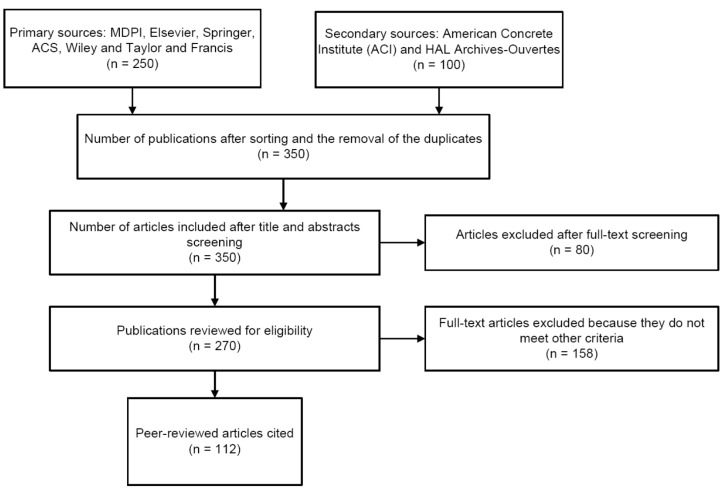
PRISMA diagram.

The review of sensors for structural health monitoring of agricultural structures was aligned with the PRISMA guidelines for systematic reviews and meta-analyses (see Figure 2). All data was sourced from published articles. The peer-reviewed data was sourced from the following primary databases: MDPI, Elsevier, Springer, and Taylor and Francis. The primary keywords were sensors, agricultural structures, health diagnosis. The inclusion and exclusion criteria was characterized by a title and abstract screening followed by a full-text and abstract screening process, which focused on the relevance of the subject to the structural health monitoring of agricultural structures.

## 2. Environmental Degradation of Construction Materials in Farm/Non-Farm Environments

### 2.1. Corrosion from Sludge and Wastewater/Manure/Sewer Channels

Agricultural structures such as greenhouses, silos, storage rooms, and general farm structures are susceptible to corrosion triggered by frequent exposure to wastewater and sludge. According to Li et al. [17], the corrosion of sewer lines in agricultural environments occurs over an extended time. The lowering of the pH defines the first phase of damage from 13 to 9—a process triggered by H_2_S dissolution and carbonation [17]. The reduction in the pH creates a conducive environment for microorganisms to thrive. Some of the most common microorganisms that thrive in this environment include acidophilic and neutrophilic oxidizing bacteria that turn sulfur-containing compounds into sulfuric acid, a key driver for acidic corrosion of steel structures and reinforced concrete. The reaction between H_2_SO_4_ and concrete generates CaSO_4_—a chemical byproduct that contributes to reinforced concrete’s weakening. Based on the concrete degradation mechanism in sewer/wastewater environments, H_2_S sensors are integral to structural health monitoring. Presently, various types of sensors have been developed for monitoring H_2_S, including ion-sensitive electrodes (ISEs), polarographic H_2_S sensors, and electrochemical detection [54]. Despite the overwhelming evidence on sulfur-oxidizing bacteria and the buildup of chemicals that trigger corrosion, there is inadequate scholarly focus on farm environments’ preventative measures.

Apart from the sulfur-oxidizing bacteria, the degradation of concrete in wastewater/sewer environments is exacerbated by algae, accelerating bio-deterioration/bio-corrosion of reinforced concrete [55]. In place of structural health monitoring in the early phases of H_2_S-induced chemical corrosion, the installation of H_2_S sensors should be explored as a first-line preventative measure. The proposal is validated by the role of H_2_S on algae and bacterial action. Moreover, accurate detection of H_2_S is critical to the inhibition of H_2_S and the elimination of any gases generated and chemically induced corrosion.

To the best of the researcher’s knowledge, electrochemical sensors’ employment to detect H_2_S in sewer and wastewater systems in farms to mitigate corrosion and structural damage is an under-explored area. Published research on H_2_S sensors has focused on the detection of H2S in biological samples (human, trout, rat, and rainbow trout plasma) rather than farm environments [54]; this means that there is a critical gap in the body of knowledge on effective prevention of structural damage in concrete structures. The lack of concerted measures introduces unquantifiable risks considering that sulfate attacks are a microcosm of the damages that might be induced by various microorganisms in different farm environments.

### 2.2. Cost of Corrosion and Material Degradation

The corrosion of metallic structures is a critical challenge in the Middle East, Asia, Europe, Africa, and the Americans due to de-icing, exposure to saltwater in marine environments, which is a risk factor for chloride-induced rebar corrosion damage. The cost implications guide the installation of sensors as a preventative measure. One study estimates that corrosion-related costs exceed £80 billion/year [56]. The costs could be higher in developing countries where monitoring practices are poorly established. The corrosion problem is ubiquitous in small and large structures such as buildings, bridges, electricity, gas, steam, AC, supply, water supply, sewage, waste management, remediation systems, and storage areas. The data presented in Table 3 demonstrate that agriculture COC is among the highest across all major economic regions as a percentage of the GDP. For example, $56 billion is lost through corrosion repair in China annually. Such expenses can be mitigated via sensor-mediated structural health monitoring. The ubiquity of corrosion provides compelling reasons why it is necessary to stall sensors in reinforced concrete structures.

The processes that led to the environmental degradation of agricultural structures provide cues on the cost-benefits of installing sensors for structural health monitoring. For example, since the environmental degradation of concrete exposed to agricultural chemicals and agricultural waste/sewer takes several years [17], it would be prudent to install the sensors in subsequent operation years. Beyond the timelines for structural degradation, exposure to different environments helps predict the risk of structural damage—concrete exposed to moisture and acid (biodegraded H_2_S and sulfate products) is less susceptible to damage than metallic structures.

Agricultural structures (such as greenhouses, animal sheds, channels, storage areas, and silos) are subjected to different environments that increase the risk of corrosion, including wastewater treatment areas, animal waste collection areas, moisture from animal feed storage areas, lagoons, agricultural chemicals such as pesticides and insecticides. Each source of material degradation poses unique risks depending on the frequency of contact and material type. For example, metallic structures are highly susceptible to corrosion while concrete surfaces are at risk of cracking, spalling, deterioration, and delamination; this means that the sensors’ design process should factor the unique environments, risk factors for material failure, and elements that might impair sensor function. The proposal is premised on the fact that the electrical components in sensors are impaired by frequent exposure to acids and moisture.

### 2.3. Water–Wood Structure Interactions

Recent experimental data confirms that water–wood interactions are influenced by physical and chemical properties of the wood [50]—the two properties are interrelated. The physical properties of wood predict the presence of various functional groups. Wood products containing lignin have the highest concentration of OH-groups (35–50), as shown in Table 4 [50]. In contrast, cellulose contains the least OH group. The presence of the OH groups predicts the level of susceptibility to water attack since the OH groups in water bond with the OH groups in lignin, cellulose, xylan, and glucomannan, which indicate the classification (soft/ hardwood). Beyond the chemical functional groups, ambient temperature and relative humidity predict the severity of water attack on wood.

**Figure 3:**
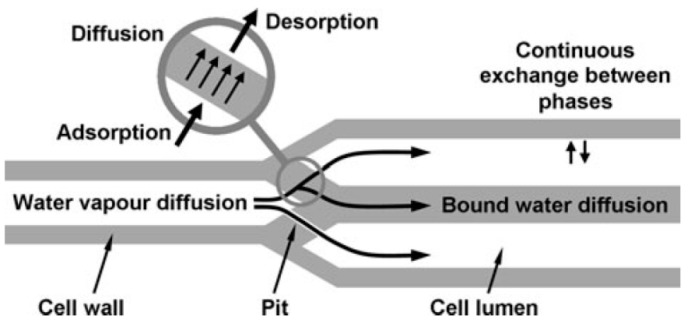
Impact of water on wooden structures [50].

The mechanism through which water weakens the strength of wooden structures is shown in Figure 3. Apart from water, termites contribute to structural damage—a factor that has informed the development of sensors to monitor the extent of termite infestation [57]. In brief, the environmental degradation of wood follows a distinct mechanism, which, in turn, validates the need for real-time evaluation of the heat, air and moisture (HAM) phenomena [58]. However, Straube et al. [58] note that the impact of current on the structural health monitoring must be considered; high voltages (>10 V) increase the risk of measurement errors.

## 3. Sensors for Health Monitoring of Agricultural Structures

**Figure 4:**
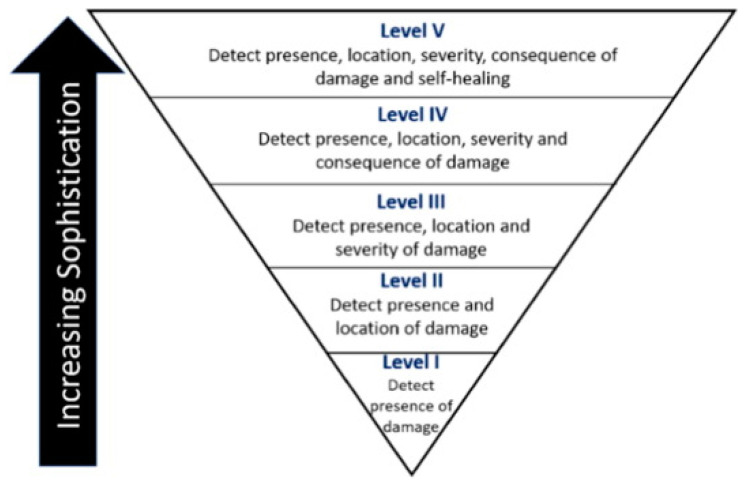
Relationship between increased sophistication of building sensors and performance [15].

The process of selecting sensors to monitor concrete structures’ structural integrity depends on the material properties, environment, age, and purpose of the structure and surface treatments [59]. For example, electrochemical sensors are appropriate for steel and metallic structures, while wireless, ultrasonic, piezoelectric, and temperature sensors are suitable for concrete structures [3,30]. Apart from the material characteristics, the identification of suitable sensors is also predicted by the level of sophistication, as shown in Figure 4. Highly sophisticated sensors provide a more reliable diagnosis of the health of the farm structures. In contrast, a decrease in sophistication translates to less accuracy—the increased sophistication reported by Mccarter and Vennesland [1] and Ha et al. [11] is consistent with Cawley’s [60] classification of structural health monitoring systems into machine condition monitoring, the global monitoring of large structures, detection of localized damage such as cracks and corrosion, monitoring of damage over large areas, and the global monitoring of large structures. Localized damage monitoring requires a higher number of sensors compared to machine learning conditions, resulting in limited specialist commercial applications.

Considering that ultrasonic, electrochemical sensors were suitable for specific types of materials, specific sensors should be developed to match the unique requirements and properties of construction materials, including composites, mass timber, self-healing materials, passive cooling ceramics, air cleaning bricks, and strand rods, and other construction materials produced from waste products. The perceived limitations inform the criteria for the selection of the existing sensors. Certain sensors cannot accurately measure hygro-thermal behavior and viscoelasticity in timber structures [61]. Other criteria for selection include replacement and the damage level.

### 3.1. Selection of Sensors for Structural Health Monitoring

The selection criteria of sensors for structural health monitoring are influenced by various factors, including the damage inspection, damage level (1–6), desired outcomes (maintenance, repair, replacement, rehabilitation, strengthening, and upgrading), and the nature of the defect. For example, most greenhouse structures are constructed using plastic materials such as PVC, LLDPE, HDPE, and D-polymer [12]. Such materials are highly susceptible to environmental degradation due to frequent exposure to UV radiation, and rain; this increases the need for real-time monitoring of the structures. Similarly, the exposure of concrete to environmental forces, continuous loading, freeze-thaw-attacks, chemical attacks, and curing conditions elevate the material’s susceptibility to failure. Even though the risk of failure can be mitigated by material modifications, including changes in the curing conditions, aggregates, water/cement ratios, and the cement type [47], concrete failure is inevitable.

**Figure 5:**
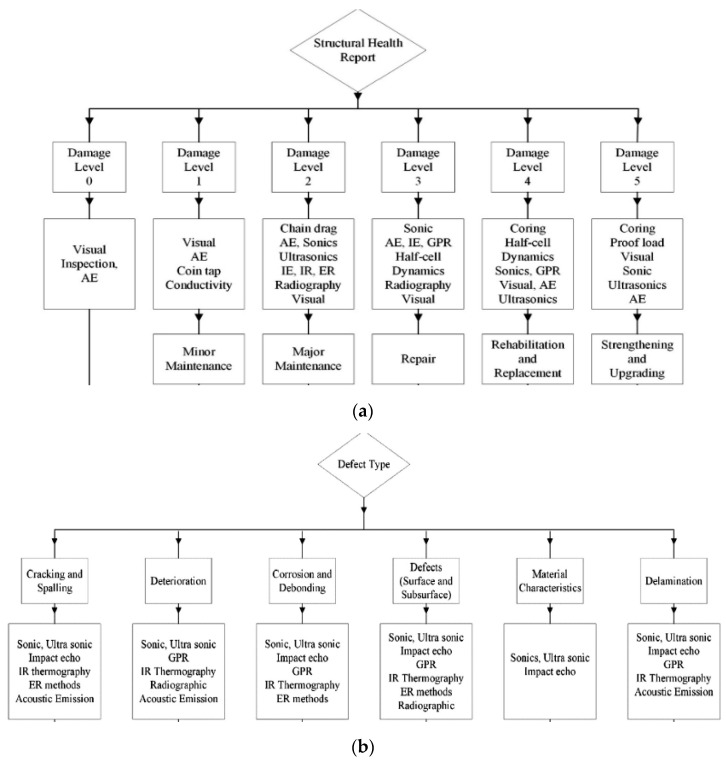
(**a**,**b**) Selection criteria for sensors based on the type of defect, level of damage, and repairs [64].

Common defects in concrete structures include corrosion, cracking and spalling, deterioration, de-bonding, surface and subsurface deformations, delamination, and material performance impairment [56,57,58,62]. The classification scheme proposed by Rehman et al. [63] (see Figure 5) suggests that ultrasonic sensors and IR thermography for structural health monitoring are suitable techniques for assessing various types of defects, including spalling, corrosion, cracking, and delamination. In contrast, acoustic emissions are only appropriate for delamination, cracking and spalling, and deterioration. The broad range of applications is augmented by the low maintenance costs and the high load-carrying capacity of ultrasonic sensors. Beyond the classification of damage and the types of repairs needed, selecting a specific class of sensors depends on the accuracy of support technologies. For example, location algorithms’ accuracy is critical to the placement of sensors in locations with a high risk of localized damage. Additionally, advances in self-powering electronics predict the utility of wireless sensors—battery-powered sensors have a limited utility [64,65,66]. Similarly, the hardiness of the materials used in sensors influences the accuracy of the measurements and functionality. Low-frequency ranges have remained a fundamental concern in the transmission of sensor signals over physical barriers.

The observations made by Rehman et al. [64] are consistent with the IAEA guidelines on NDT in concrete structures [67]. However, the latter study suggests that other factors should be considered when selecting NDT methods and sensors, including the path length, the time required for the pulse to traverse the sample, and the frequency. The latter proposals illustrate that other critical considerations influence the sensors’ selection; these include the material properties, risk of errors, shape (regular or honeycombed concrete), and size of the specimen. The probability of error in sensor measurements is a critical limiting factor because structural health monitoring data inform repairs, maintenance, rehabilitation processes. According to Ndagi et al. [68], the measurement error in ultrasonic sensors is 7%. The errors might be exacerbated by environmental conditions such as air humidity [66], common in farms. Considering that the environmental conditions cannot be regulated, the functionality and utility of wireless sensors in farm structures could be severely impaired by humidity and moisture.

On a positive note, the risk of bias attributed to measurement errors can be mitigated by adopting complementary NDT techniques [67]. In contrast, Chakraborty et al. [69] noted that the probability of accurate detection was 100% for ultrasonic sensors. The two studies demonstrate the context-specific factors that predict sensor accuracy. In the latter study, embedded sensors were able to detect damage in hard-to-reach sections of the concrete superstructure [69]; this is distinct from the approach adopted by Ndagi et al. [68], where ultrasonic pulse velocity was applied on the surface. A fundamental concern is a mismatch between theory and practice. The theoretical guidelines for selecting NDT sensors proposed by Rehman et al. [64] and IAEA [67] are not universally accepted. Engineers often select techniques that suit specific contexts; this helps explain why ultrasonic sensors are not extensively employed in structural health monitoring in commercial agriculture.

### 3.2. Sensors for Structural Health Monitoring

#### 3.2.1. Electrochemical Sensors for Monitoring Chemical Corrosion

The widespread risk of environmental degradation reinforces the installation of wireless and non-wireless electrochemical sensors for real-time corrosion monitoring. The corrosion of steel, concrete reinforcements, and degradation of plastic materials remain a complex challenge in farm environments, despite the development of advanced methods of passivation [70,71], cathodic protection [72,73], galvanizing, corrosion inhibition [74] and fiber-reinforced polymers for preventing corrosion [75], and development of metal stabilizers and UV absorbers to limit the impact of UV radiation [12]. The failure of existing mitigation methods could be due to the frequency of exposure. For example, wooden and concrete agricultural buildings for poultry and dairy production are continuously exposed to liquid waste from dairy and poultry manure, which diminishes the rigidity and strength of wood [50] and corrodes the metal reinforcements in concrete. On average, the building structures are corroded at a rate of 0.0474–0.0094 g/cm^2^/month [5] (see Table 5). The corrosion rate could be higher depending on humidity, temperature, and the presence or absence of a protective coating. Apart from the chemical-induced corrosion, the temperature is a critical variable because it triggers greater magnitudes of flexural stress in the structural columns [76], which might result in structural failure.

The thermodynamics of heat treatment elevates the possibility of corrosion because purified metals and alloys are thermodynamically unstable; this was demonstrated by exposing AISI 444 and 304 stainless steel variants to corrosive environments [77]. In addition to material thermodynamics, the metal’s electrical resistivity has a direct impact on the probability of corrosion [3]. Considering that corrosion is unavoidable due to the thermodynamic processes involved in metal purification and fabrication of structures, proactive monitoring of structures for corrosion signs is a practical alternative.

Other critical parameters should be taken into account during the selection of appropriate sensors for structural health monitoring; these include stiffness, moisture, density, dynamical properties, deformations, decay, cracks and delaminations, inhomogeneity, and modulus of elasticity The data in Table 6 illustrates that the material parameters influence the selection of an appropriate method for structural health monitoring [78]. For example, electrical resistance, hygrometric, and dielectric methods were best suited for assessing moisture damage, including material decay. In contrast, self-healing materials were only exposed to insect damage. However, the parameters of interest might not be an accurate predictor for the monitoring technique, especially for building structures that integrate different materials such as concrete and steel (reinforced concrete), composites, and polymers. Additionally, engineers have to consider the cost, sustainability, and accuracy of the monitoring technique. In brief, the parameter of interest is not always a primary consideration.

Even though electrochemical sensors are appropriate, the following considerations should be observed in the selection process; electrical resistivity, which predicts the de-passivation rate for steel and corrosion risk. Additionally, there is a strong relationship between the accurate assessment of the rate of corrosion and the electrode potential [3]. In a majority of applications, caramel electrodes are used as the reference electrodes for the best accuracy. Considering that the risk of probable corrosion varies widely across different farm environments (dairy, poultry, and swine manure) [5], the type of electrochemical sensors used should be customized to improve the accuracy in health monitoring.

#### 3.2.2. Electrical Capacitance Tomography (ECT) Sensors

The function of electrochemical sensors is augmented by the installation of ultrasonic sensors, which measure the probability of crack propagation using ultrasonic pulse amplitude (USPA), acoustic emission technique (AE), digital image correlation (DIC), and displacement [47]. Each type of sensing technique offers unique advantages and limitations depending on the intended use and the environment. For example, the suitability of ECT sensors in monitoring the structural health of silos was demonstrated by Niedostatkiewicz et al. [79]—ECTs yield accurate 2D data on the solid bulk flows within a silo. On the downside, the rate of degradation and damage was not constant—it was influenced by the pH level, presence of chemical fluids, and concentration of the acidic and ionic ions (Cl^−^ and OH^−^) [80]. The challenge impacted the reliability of ECT, especially in environments with fluctuating pH levels; this means that ECT sensors should be programmed to match the unique environments where the technology is applied. Considering that ECT was best suited for corrosion monitoring and less ideal for assessing changes in bulk solids’ movement in silos, potential applications in farms are limited. The underlying electrochemical principles were employed in the development of ECT technology for analyzing permittivity in bulk solids.

**Figure 6:**
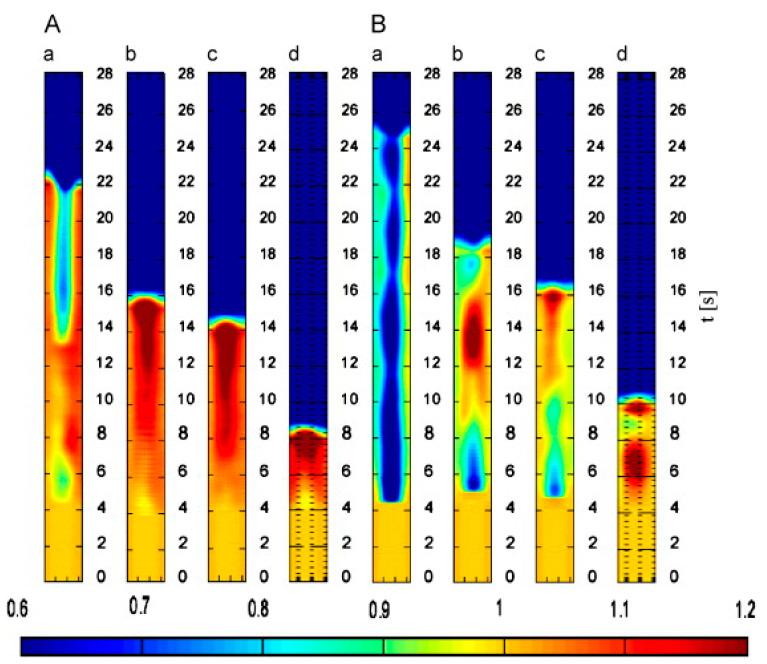
Electrical capacitance tomography (ECT) two-dimensional visualization of silos under different capacities; (**A**) initial loose sand (**B**) initial dense sands [79].

The electrochemical sensor technology also forms the foundation for electrical capacitance tomography (ECT)—a reliable technology for real-time monitoring of granular changes in silos [79]. Niedostatkiewicz et al. [79] reported the utility of ECT in determining the changes in permittivity—with an accuracy of 3–5%. On the downside, the technique’s accuracy was proportional to the electrical conductivity of the target materials. Lower electrical conductivity translates to better measurements. The thermal imagery of the granular distribution in different silos is shown in Figure 6. Considering that the silos under investigation were loaded with sand, it would be interesting to determine whether similar observations would be made in agricultural silos. Recent studies suggest that the technology was appropriate for structural health monitoring of silos that store agricultural produce, especially in solid grain form. Both Grudzien et al. [28] and Zurovec et al. [81] monitored granular flow changes in corn and wheat silos using ECT; it was deduced that the technique was appropriate to help obtain critical information about the behavior of bulk solids.

The real-time monitoring of the flow profile is vital because the uneven distribution of solid materials might result in the unequal distribution of the grain moisture content, axial and hoop forces, pressure ratio, and internal friction angle [6]. The relationship between solid bulk flows and different types of silo obstructions, including noise and vibration, segregation, ratholing, and arching, which result in the buildup of overpressures on the hopper walls, reinforce this argument. Although positive outcomes were reported by both Grudzien et al. [28] and Zurovec et al. [81], the utility of ECT in structural health monitoring is less explored. The monitoring of the discharge process might not provide an accurate assessment of the presence of structural defects because the sensitivity maps and visualizations are less detailed, as shown in Figure 6. The challenges attributed to the spatial resolutions were noted by Grudzien et al. [28], who attributed the phenomena to the number of electrodes and the non-linear nature of the electrical field. Current technologies do not provide a practical solution to the challenge because image reconstruction techniques yield images that difficult to interpret. Apart from the technical challenges, the technique is time-intensive; hence, not suited for time-sensitive analyses. The imaging-related constraints [28] suggest that ECT is only appropriate for the analysis of the granular flow/discharge process of bulk material in silos.

Considering that ECT had limited functionality in the structural health monitoring in agricultural silos, novel ultrasonic, wireless, and piezoelectric sensors should be employed to ascertain the extent of the corrosion, cracking and spalling, deterioration, de-bonding, surface and subsurface deformations, and delamination on the silo walls. One of the novel developments was presented by Pal et al. [82]—the study employed capacitance sensors for level measurement in hoppers/silos [82]. A key shortcoming was material-specific reliability—the technique was only demonstrated in metallic silos. Further research is necessary to demonstrate the utility of the technique in concrete silo structures. A fundamental question is whether it was economical to embed both ECT and ultrasonic/fiber optic sensors on silos. In theory, it would be imprudent to focus on both bulk grain flows and the structural integrity of silo—the latter criteria takes precedence because it is indispensable to the durability of silo structures; this means that information on bulk flow can be disregarded if the cost is a primary consideration.

#### 3.2.3. Ultrasonic Sensors for Monitoring Crack Propagation in Concrete

Ultrasonic sensors are appropriate for monitoring crack propagation in concrete structures because sonic waves are sensitive to the micro and mesostructures, particularly impedance changes and structural geometry [47]. The utility of ultrasonic sensors in timber and greenhouse structures is less well established perhaps due to the longevity of the structures—greenhouses and timber structures are less durable compared to concrete.

Time considerations inform the method’s practicality—quick scanning is impractical because manual ultrasonic sensors are mounted on the target surface and operated manually through a time-intensive process. Alternatively, the sensors might be embedded in the concrete structure even though the embedding of the sensors reduces the time and resource requirements. The integration of sensors into concrete might limit the sensitivity of the sensors; this is because the ultrasonic sensor ability is directly proportional to the compressive strength of the material [83]. The need to select materials with an appropriate bandwidth might translate to a tradeoff with the mechanical properties.

The link between compressive strength and ultrasonic sensitivity is calculated using a linear mathematical relationship presented by Chakraborty and Katunin [84]. The formula suggests that the measurement of the skimming longitudinal wave velocities and Rayleigh waves are reliable predictors of material damage; this is because the speed of sound is reduced or enhanced by the microscopic arrangement of the atomic particles and elasticity. The two properties predict the thickness of a material and the extent of crack propagation. The relationship between the two core variables is derived using Equations (1) and (2), where *E* is the material modulus, rho- density. At the same time, the Poisson’s ratio is denoted by n. The sound velocity for the Rayleigh and the longitudinal waves is represented by *V_r_* and *V_l_*, respectively [84]. The two equations draw a relationship between the material elastic properties, wave velocities, and the density of the material, which contributes to the estimation of the attenuation and the diffraction of the waves. The ultrasonic waves should be derived from a high energy source with a low frequency based on the linear relationship.

(1) $$V_{r}=\;\frac{0.87+1.12n}{1+n}\sqrt{\frac{E}{2\rho \left(1+n\right)},}$$

(2) $$V_{l}=\sqrt{\frac{E\left(1-n\right)}{\rho \left(1-n\right)\left(1-2n\right)}},$$

From another point of view, the mathematical relationship is not representative because the nature of the load, age of the structure, presence or absence of steel reinforcements, and the volume of the material impact the structural material density. Multiple studies have demonstrated that the age of concrete predicts material deterioration and sensor function [85,86]. However, this is only true for aged structures due to gradual exposure to rain, moisture, and corrosion inducing elements. The compressive strength increases in new structures [87] while the water absorption rates diminish over time.

**Figure 7:**
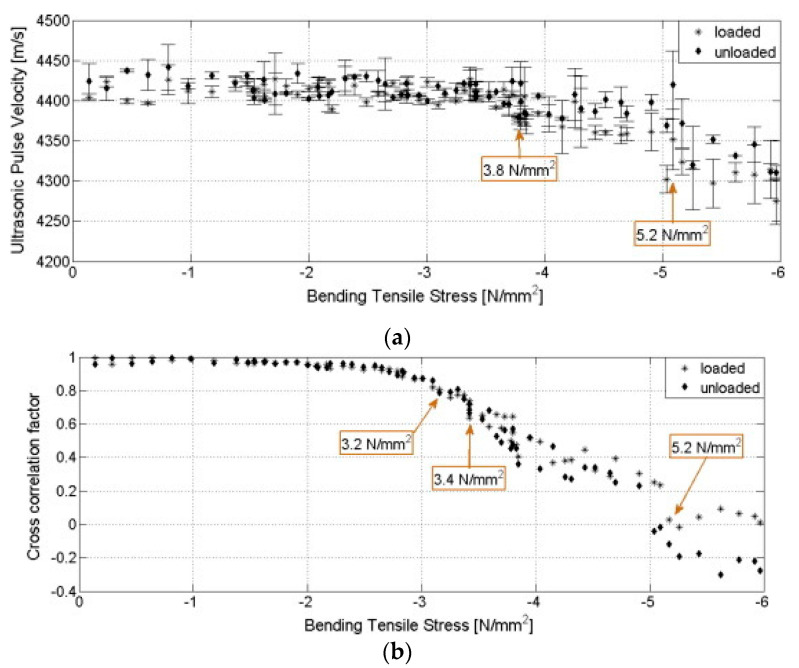
Ultrasonic pulse velocity (USPV) (**a**), cross-correlation factor of signals from cyclic loading/unloading (**b**) [47].

Silo concrete structures have a higher density [88,89] compared to stand-alone and empty concrete structures. The observation is in line with Janssen’s theory on the impact of density on the structural pressure [90]. Apart from the loading, the age elevates the velocity of the ultrasonic waves, possibly due to the concrete’s maturation and the changes in the incident angles [85]. The impact of structural loading on sensor function was also confirmed by Wolf et al.’s [47] experimental data, which suggested that the ultrasonic pulse velocity was inversely proportional to the load—an increase in the material’s load due to crack growth and widening translated to a drop in the ultrasonic pulse velocity. Cracks in the concrete microstructure are demonstrated by the noticeable changes in the ultrasonic pulse velocity (USPV) and the cross-correlation factor of signals from cyclic loading/unloading, as illustrated in Figure 7 [47]. In particular, visible changes are observed at 5.2 N/mm^2^, 3.8 N/mm^2^, 3.4 N/mm^2^, and 3.2 N/mm^2^.

**Figure 8:**
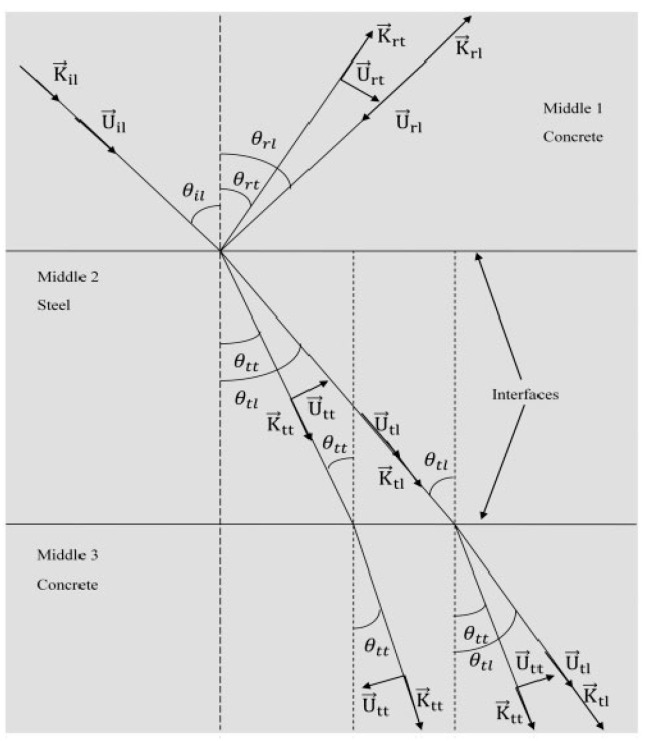
Reflection and transmission of ultrasonic waves on high-performance and ordinary concrete and steel [85].

A comparative analysis of the transversal propagation velocity, reflection, transmission, and longitudinal propagation velocity, of high-performance concrete, steel, and ordinary concrete (density = 2311.69 kg/m^3^) in Figure 8 shows that the age of the material had a pronounced impact on the material properties. Since ordinary and high-performance concrete composition varies across different regions, the UPSV patterns would be inconsistent. The variable composition of concrete has been widely documented in research. For example, Rasol [21] enriched concrete with silica fumes to increase the mechanical strength, while Bassuoni and Rahman [91] reported better porosity in fly-ash-concrete specimens compared to the controls. In the latter case, the samples’ porosity had a domino effect on the mechanical and physical properties. The impact of the material modification on physical, chemical, and mechanical properties illustrates that distinct cross-correlation factors of signals could be reported from two high-performance concrete samples drawn from different locations. Local materials that satisfy the local construction requirements could limit the standardized application of ultrasonic sensors, which rely on piezoelectrics. According to Maier, the concrete microstructure modification has both direct and indirect effects on the piezoresistive properties [92].

The longitudinal propagation velocity and transversal propagation velocity for a 45-day old high-performance concrete (density = 2409.33 kg/m^3^) were 4209 and 2420 m/s, respectively [85]. In contrast, the longitudinal propagation velocity and transversal propagation velocity for ordinary concrete at day zero were 3899 and 2241 m/s, respectively [85]. Wolf et al. [47] reported comparable ultrasonic velocities in concrete structures embedded with ultrasonic sensors. The experimental data reaffirms existing theories on the direct relationship between the concrete’s age and the ultrasonic pulse velocity.

Based on the findings presented by Huang et al. [83], Raach et al. [85], and Chakraborty and Katunin [81], the sensitivity of the ultrasonic sensors for structural health monitoring of concrete structures would increase in line with the age of the concrete structure. Beyond the age of the material, other parameters had a direct influence on structural health monitoring, including the cement quality and mechanical properties [47,83,84,85]. The ultrasonic waves’ speed is enhanced by the presence of closely arranged steel reinforcements, temperature, humidity, water to the cement ratio, and the arrangement of the reinforcements [83,84,85]. The presence of closely packed reinforcements translates to better movement of the ultrasonic waves, given that metals have flexible atomic structures and lattices.

Even though ultrasonic sensors’ deployment is recommended based on the positive outcomes reported in real-life and simulated conditions, there are critical technical drawbacks that should be addressed. First, embedded sensors collect diagnostic data only. Alternative signal processing techniques such as the first arrival, time delay, Hilbert transform of ultrasonic wave are applied to interpret the raw findings [84]. On the downside, the technique’s sensitivity is questionable, especially at low frequencies, which are appropriate for ultrasonic measurements. The accuracy of the signal processing techniques is also impacted by noise. The core limitations of the ultrasonic sensors illustrate that advanced methods of signal processing are required. New and highly sensitive methods have been explored, including the autoregressive model [84] and acoustic emissions [47].

#### 3.2.4. Fiber Optic Sensors for Crack Monitoring

**Figure 9:**
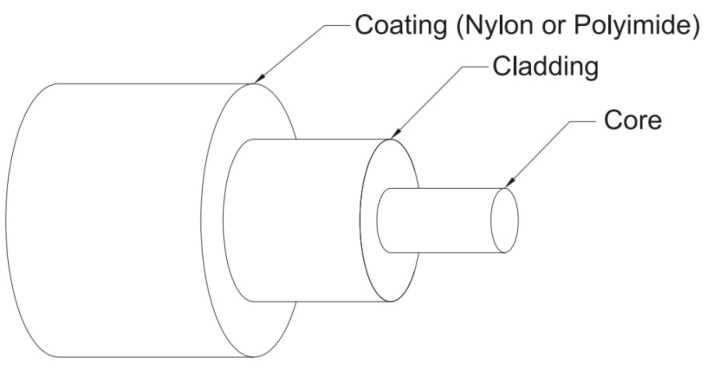
The basic configuration of a fiber optic sensor for structural health monitoring [9].

Various types of fiber optic sensors have been deployed for structural health monitoring applications [93]. The most effective include evanescent field-fiber loop ring-down (EF-FLRD) techniques [35], optical [94], and distributed fiber optic strain sensors [9] for water and moisture detection in concrete. As noted in the preceding sections, the choice of the sensors is predicted by the functionality, application, and reliability. For example, EF-FLRD is a reliable technique for measuring the changes in the water and moisture content—the feature is augmented by the high reversibility in water–dry–water–dry duty cycles and detection sensitivity limit (approximately 0.12 mL) [35]. The unique benefits associated with EF-FLRD justify the choice of the sensor in place of polymer-coated fiber Bragg grating (FBG) water sensors. The accuracy of the fiber optic sensors is attributed to the configuration of the components depicted in Figure 9. The main components include coating, cladding, and the core (source of light transmission) [9]. The cladding component helps to convert the core into a waveguide, while the polyimide or nylon coating offers protection and resistance against the applied forces.

Even though all fiber optic sensors have a similar configuration, López-Higuera et al. [16] noted that the accuracy and reliability of the measurement were influenced by spatial distribution of the measurand, fiber optic sensors (optical, point, integrated, quasi-distributed, and distributed), type of technology (fiber Bragg grating, Raman scattering, and SOFO), bandwidth speed, accuracy, resolution, and the type of the transducer [16]. The observations made by López-Higuera et al. [16] are in agreement with Kaya et al.’s [35] research on the reproducibility of fiber optic sensors for structural health monitoring. In the latter case, the hand fabrication process predicted the sensitivity of the sensor lops and heads. The absence of a standardized and automated fabrication process contributed to variations in the total optical loss, length of the fiber loo, and the sensor head. Secondary issues include Raman, Brillouin, and Rayleigh backscattering. On a positive note, the constraints can be resolved by integrating two fiber optic sensors (reference and measurement sensors) [76]. The fiber optic sensor measurements are less susceptible to electromagnetic interferences and are characterized by higher sensitivity and low costs. Additionally, the tests can be undertaken in specimens of different weights and sizes [15].

There is limited empirical research specific to farm structures. A majority of the research studies investigated the suitability of the sensors in general construction operations [9,16]. The lack of conclusive data is a critical limiting factor because the risk of structural damage is proportional to the building’s purpose. Bridges and silos are subjected to frequent loading and unloading, which increases the risk of failure. The data confirm a causal relationship between the crack tip opening displacement (CTOD) and the crack’s length at peak loads [94]. The embedded fiber optic sensors measured the crack length by correlating the light intensity variation of the speckle patterns with the applied strain resulting from mode re-distribution. In brief, fiber optic sensors offer key benefits in structural health monitoring; nonetheless, further research is necessary to establish the utility of dual fiber optic sensors in farm structures before widespread use.

#### 3.2.5. Fiber Bragg Grating (FBG) Sensors

The Fiber Bragg grating sensors are based on similar principles as the fiber optic sensors (FOS); this makes them ideal for structural health monitoring of concrete, wood and greenhouse structures exposed to moisture and water [95] compared to the traditional fiber-optic sensors (FOSs) [22]

The water/ moisture is detected using a moisture-sensitive polymer, which expands upon exposure to water resulting in a shift in the fiber Bragg’s wavelength [96]. Moisture changes are quantified by the variations in the refractive index attributed to the absorption of the moisture. according to the mathematical relationship presented in Equation (3), where Λ, *n_eff_*, and *λ_B_* represent the index modulation, the effective index of refraction, and the center wavelength of the resonance condition [22,23,95].

(3) $$\lambda_{B}=2n_{eff}\Lambda$$

One of the most remarkable improvements includes multiplexing capability (wavelength division multiplexing (WDM) technology) [23], immunity to electromagnetic noise [24], and quasi-point sensing that is least impacted by wavelength light path disturbances [22]; these features are absent in the conventional FOS. The functionality of the FBG sensors has been reliably demonstrated in a wide array of applications. For example, Zhang et al. [95] employed the technique to monitor concrete arch damage. The sensors yielded critical time-series data that was instrumental in determining the crack time through complex empirical mode decomposition (EMD) and Hilbert–Huang transform (HHT). The FBG sensors were reliable in the monitoring of structural damage in woodworks by Falciai et al. [97] at high resolutions. The sensors accurately detected panel deformations and structural changes following the removal of the cross-beam panel. The positive outcomes indicate that FBG sensors can accurately monitor the strength and performance of wooden structures in farms. Alternatively, FBG sensors can be integrated with resistive sensors and termite sensors to improve performance [57,98] and limit errors. Despite the proven effectiveness of the sensors on wooden structures, voltage regulation remains a key concern. Higher than normal current might trigger the migration of salts and uneven heating of the wooden surface [77]; the net effect is inaccurate measurement.

**Figure 10:**
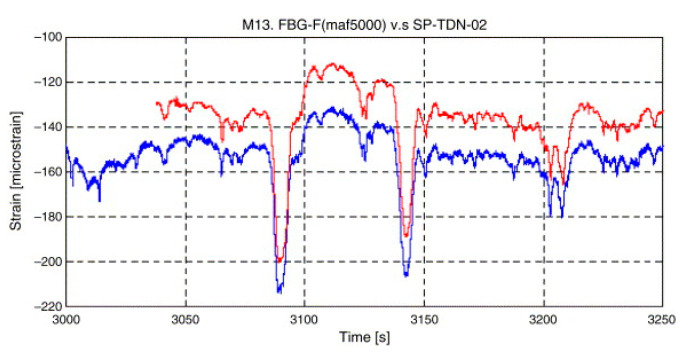
Comparative analysis of the Fiber Bragg Grating (FBG) spectrum (red) and the traditional strain gauge measurements (blue) used to monitor the structural integrity of suspension bridges [22].

A key challenge associated with FBG sensors is reliability given the mechanism is influenced by the point pasting method—poor placement of the sensors resulted in a poor prediction of the initial crack, crack evolution, and the arch ruins [95]; this illustrates the role of experimental setup on the reliability of the FBG output. Despite the methodology related constraints, practical case studies of FBG technology affirmed its reliability. For example, Chan et al. employed the technique in the structural health monitoring of Hong Kong’s landmark Tsing Ma bridge—one of the world’s largest suspension structures [22]. The research confirmed a close correlation between the FBG results and the measurements made using traditional methods of structural health monitoring, as demonstrated in Figure 10.

Considering that the output signal is least impacted by the losses in the connecting couplers and the fibers and the output is comparable to other structural health monitoring methods [22], the FBG could complement ultrasonic sensors in the monitoring of the structural health of concrete structures. The case for FBG sensors in structural health monitoring is further reinforced by the unique wavelength of each signal—a factor that facilitates easy monitoring ad multiple simultaneous measurements using wavelength-multiplexed FBG sensors [22,23]. Additionally, comparative experimental studies have demonstrated that FBG sensors are better compared to RSG sensors, especially in the monitoring of concrete subjected to sustained loading conditions [24]. The accurate assessment of strain affirms the potential utility of FBG in agricultural silos, which are subjected to frequent loading and unloading.

Practical constraints include the lack of extensive case studies demonstrating the utility of FBG sensors in farm environments. Such concerns are valid because positive assessments drawn from suspension bridges might not accurately predict FBG sensors’ reliability in farm structures. The two types of building structures were subjected to different degradation inducing forces. Similarly, structural differences between bridge tunnels and silos could complicate the accurate assessment using FBG sensors. The experimental schematics presented by Lai et al. [23] demonstrate that FBG sensors need to be installed on the walls’ interior and exterior sections, which is impractical for packed bulk grain silos. The placement of sensors on the exterior sections of the silos could result in less reliable measurements. Despite the potential technical limitations, the practical benefits such as immunity of the FBG sensors to electromagnetic noise, effective measurement of strain in pre-stressed concrete and moisture changes in operational structures validate the employment of the sensors in agricultural environments.

#### 3.2.6. Piezoelectric Sensors

In contrast to the electrochemical and ultrasonic sensors, the functionality of piezoelectric sensors is limited to pressure, temperature, acceleration, strain, or force sensing [86,87]. The testing technique exploits the relationship between Young’s modulus, material density, and harmonic amplitude. The piezoelectric sensors offer similar NDT capabilities as ultrasonic and hydration heat-based monitoring methods. However, the three techniques are differentiated by cost, reliability, accuracy, fast measurement, and ease of use. Ultrasonic sensing equipment is expensive compared to the low-cost piezoelectric sensors, while the hydration heat-based monitoring method yields less reliable data [86]. The cost benefits of piezoelectric sensors are reinforced by the small dimensions and ubiquity of materials with piezoelectric properties. For example, Lezgy-Nazargah et al. [99] developed a reliable 5 cm × 5 cm × 5 cm piezoelectric sensor from silica, resin, Portland cement, and polymeric fibers. A key advantage of the fabrication process is flexibility—the constituent materials can be modified to suit local requirements and needs. In place of composites made of silica, resins, and cement, Talakokula et al. [100] developed a fly ash-based geopolymer concrete piezoelectric material. Other materials that can be used in the synthesis process include multi-wall carbon nanotubes, graphene, and carbon fibers [101] capable of transforming applied strain/pressure/force into electrical resistance.

The ability to locally fabricate sensors contributes to significant cost savings compared to the purchase of ready-made sensors made using special fabrication technology. The long-term monitoring of the material is impacted by the bonding defects between the host structure and the piezoelectric sensor patches and the degradation of the transducers’ electrical and mechanical properties [27]. Despite the technical constraints, positive outcomes have been documented in selected case studies. For example, Chen et al. confirmed that the technique was appropriate in early-age strength monitoring [86]. At the same time, the reliability of the piezoelectric sensors in a broad range of real-life applications was validated by Park et al. [27] and Lezgy-Nazargah et al. [99]. From the researcher’s point of view, the technical constraints of piezo-materials could be resolved with the advances in technology, leading to the widespread use of piezo-materials in agricultural settings.

#### 3.2.7. Wireless Sensors

Wireless sensors have been widely applied in structural health monitoring of concrete structures [37,66,86,102,103]. Apart from concrete, wireless sensors have been extensively applied in greenhouses for smart farming applications. The automation of greenhouse structures using wireless sensors has facilitated the remote monitoring of the local climatic conditions in different sections of a greenhouse [104]. The cumulative effect includes better productivity and reduction of agricultural losses. Similar benefits accrue from the installation of sensors in timber structures. The monitoring of timber in agricultural environments is a priority considering that the material is susceptible to water-induced degradation, which favors the growth of fungi [1,98]. Key advantages include the possibility of long-term monitoring and accuracy.

In contrast to other types of sensors, the utility of these sensors is reinforced by the ease and cost of installation, functionality, and durability, and accuracy of the measurements facilitated by the internet of things [44]. Abdulkarem et al. estimated that the cost of installing and maintaining wireless sensors is one-tenth of the traditional sensors [66]. The compelling advantages reported by Abruzzese et al. [44] are in line with Abdulkareem et al. [66] appraisal of the challenges, opportunities, and future potential of wireless sensor systems. In both studies, the utility of wireless sensors was established in different construction environments.

A real-world case study of wireless sensors in the structural health monitoring of Basilica S. Maria di Collemaggio established that the wireless sensors were ideal for monitoring of slowly-varying phenomena such as the impact of seismic shocks [102]. The findings reported by Federici et al. [102] are collaborated by Keller et al. [45], who accurately analyzed the thermal/temperature-induced stress that was induced by temperature fluctuations in a steel structure. The experimental observations were closely aligned with the predicted values. The difference between the reported and theoretical values was 0–120 MPa—a difference that is not statistically significant. The correlation between the two sets of values affirms the accuracy and reliability of wireless sensors.

The two case studies do not yield conclusive evidence because the structures served specific functions and were exposed to unique environmental forces, which are absent in other areas. For example, Federici et al. [103] observations were confined to a single location (Basilica S. Maria di Collemaggio); this means that the impact of distance, structural interferences, and terrain on the structure was not adequately delineated. Even though the findings and observations made by Abruzzese et al. [44], Keller et al. [45], and Federici et al. [102] are not conclusive, they offer useful insights on the application of wireless sensors in structural health monitoring in various environments.

Similar to other types of sensors, there are critical impediments to the widespread application of the sensors, including design challenges for long-term application and customized control algorithms. Other constraints include the possibility of data flooding in wireless sensor networks (WSN) [102,103], poor fault tolerance of the wireless sensor network, potential power interruptions, and lack of seamless communication between the central devices and the peripheral sensors [44]. The listed drawbacks are but a microcosm of the potential challenges that might arise from wireless sensors’ widespread use. Based on available evidence, the benefits of wireless sensors in agricultural environments do not outweigh the critical technical constraints that have largely remained unresolved.

The need to adopt a cautious approach to wireless sensors’ deployment is grounded on recent empirical data. Both Abruzzese et al. [44] and Abdulkarem et al. [66] concur that the novel technologies that facilitate the deployment of wireless sensors hold great promise and peril. A key impediment to the future application is information asymmetry—the shortcomings of wireless sensors in structural monitoring are not well documented and resolved through research and development (R&D); this also applies to the employment of the sensors in analyzing the moisture content in timber structures. The assessment of the moisture content in timber structures is necessary due to the high permeability of wood and the limitations of the surface treatment methods [1].

In the latter case, critical parameters such as the ideal sampling locations, sampling protocols, and the nature of the structure were not well defined [105]. The selection of appropriate locations is justified because cantilever, beams, girders, long and short columns are subjected to varying tension and compressive forces. The maximum tension for short columns is 97 MPa; this contrasts with 21 MPa for the girders. Similar differences were reported in the optimal compressive strength [76]. The main challenge is linking high exposure to compressive and tension forces and the risk of structural damage, given that each component is reinforced to handle different loads. In brief, there is inconclusive evidence on the utility of wireless sensors in different environments, structural applications, and designs.

The lack of conclusive evidence could be attributed to wireless sensors’ limited application relative to traditional methods/conventional sensors in concrete and steel structures. Since the shortcomings are poorly delineated, the decision to install wireless sensors in structural health monitoring and related applications should be guided by existing empirical data on the data platforms, data acquisition specifications, embedded computing specifications, data memory, wireless channel specifications, and simulations. The need for comprehensive data before deployment is supported by wireless sensors’ susceptibility to damage caused by strain and temperature interdependence—a phenomenon that is influenced by Brillouin and Rayleigh backscattering [76].

On a positive note, some of the constraints associated with wireless sensors could be mitigated by the emerging technological solutions. For example, even though the wireless sensors’ accuracy remains unknown due to the absence of reliable data, the wireless sensor approach enables engineers to embed sensors with different capabilities. According to Riggio and Dilmaghani [61], wireless sensors could be embedded with fiber optic and other types of sensors. The ability to integrate different sensors reinforces the utility of the sensors in a broad range of structural applications. Additionally, the ideal location/sampling locations could be determined using numerical simulations; this approach was employed by Castro-Triguero et al. [105,106] to address material uncertainty and identify an ideal location for placing the wireless sensor using a sensor placement algorithm, especially on solid elements, shells, plats, beams, bars, and springs. Nonetheless, the algorithm is limited because some of the proposed DOF locations cannot be measured, given they are located on interior sections or bends. Considering that the DOF locations are not useful, they are deleted from the full modal matrix. The latter case demonstrates the limitations of smart and intelligent systems for wireless sensors.

**Figure 11:**
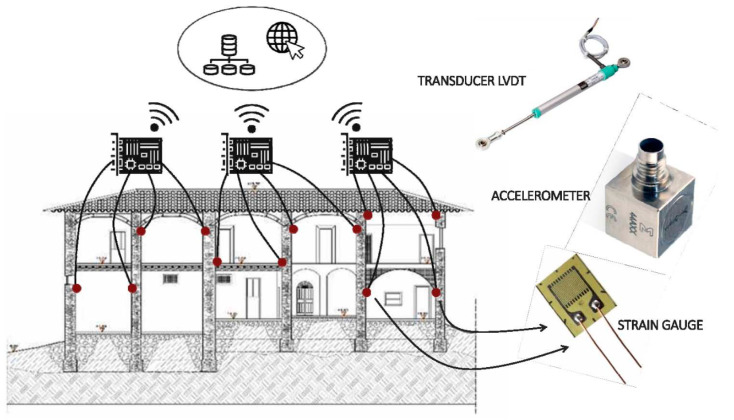
Wireless sensors for structural health monitoring [44].

From an engineering point of view, location-related challenges should not impede wireless sensors’ employment, given that certain forms of material degradation such as chloride attacks and carbonation are localized to specific sections of the superstructure [30]. Additionally, new and responsive algorithms provide data on the ideal locations for sensors, while innovations in sensor technologies mitigate the impact of power interruptions. For example, Hasni et al. [65] reported the application of battery-free wireless sensors. The self-powered wireless sensors exhibited comparable performance as other externally powered wireless sensors. The technological progress made so far suggests that power is no longer a limiting factor to wireless sensors’ deployment. However, new technology should help broaden the harvesting of electricity from other sources part from strain energy. Advances in technology have also facilitated the integration of different components such as deformometers, accelerometers, strain-gauges (see Figure 11), crack meter, inclinometer, sink, and node gateways [44,102]. The multisensory approach’s utility is supported by Federici et al. [102] research on the Basilica S. Maria di Collemaggio. The study employed various sensors to monitor damages attributed to the seismic shocks and confirmed that each type of sensor was reliable. The positive outcomes show that advances in technology have facilitated the application of wireless systems in a wide array of applications.

#### 3.2.8. Self-Sensing Concrete

There are two principal forms of self-sensing concrete: intrinsic and non-intrinsic self-sensing concrete; each offers unique structural health monitoring benefits. The non-intrinsic sensors feature multiple technologies, including optical fiber sensors, shape memory alloys, piezoelectric ceramics, electrochemical sensors, strain gauges, and conductive polymer composites [92,107]. The integration of different types of technologies has mixed benefits. On the one hand, it introduces new challenges in signal acquisition and processing, technical maintenance, and production. The sensitivity of the technology is a barrier to mass production, which, in turn, predicts the market prices. Scarcity of the self-sensing concrete translates to higher purchase prices. On the other hand, it offers practical benefits, especially in terms of improved structural and mechanical strength [108], cost-friendly integration of smart technology, and lifelong monitoring of the structural health. The practical benefits of self-sensing concrete were demonstrated by D’Alessandro et al. [108] after integrating carbon nanotubes into the cement mix to form nano-modified cementitious materials. According to D’Alessandro et al. [108], the modified cement (0.75% multi-wall CNTs) exhibited better electrical conductivity, loading, and unloading, and self-sensing ability compared to the unmodified materials.

**Figure 12:**
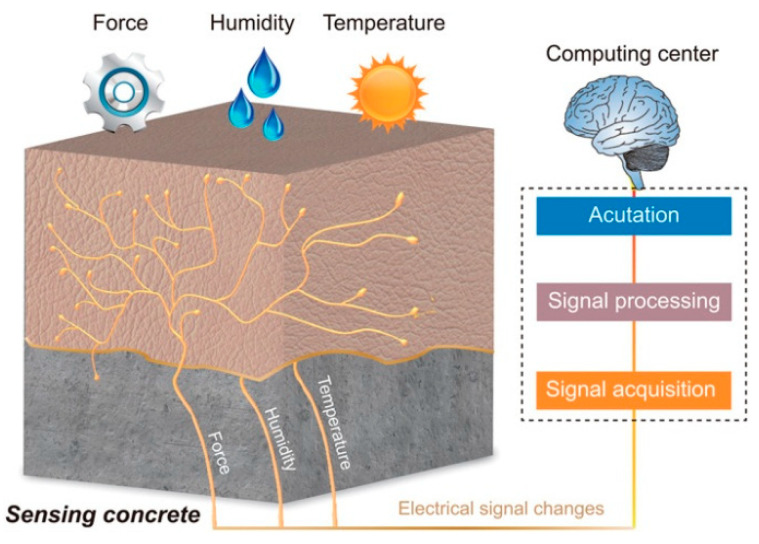
Key features of self-sensing concrete [107].

The need for self-sensing concrete in farm structures is grounded on the drawbacks of stand-alone wireless, fiber optic, and electrochemical sensors. Even though these sensors have been extensively incorporated into new, old, and historical buildings, there is a myriad of practical constraints ranging from cost, technical requirements for installation, operation, and interpretation of data, limited power requirements, unsustainable maintenance, poor accuracy in extreme weather conditions, and incompatibility with selected building materials [107]. The barriers and challenges can be mitigated by installing self-sensing concrete, which is embedded in the concrete microstructure, as demonstrated in Figure 12. Based on the illustration, self-sensing concrete mimics a human’s central nervous system by accurately detecting force/pressure, humidity, and temperature changes using various sensors for signal acquisition, processing, and signal actuation. A major constraint is the absence of conclusive data on the utility of self-sensing concrete in different farm structures. Additionally, existing research suggests that certain forms of self-sensing concrete suffer from the same technical challenges as stand-alone sensors, including impaired performance under high moisture environments [92]. Other studies have suggested that structural reinforcements impair performance; it would be non-value adding to install self-sensing concrete from a cost perspective if the traditional barriers and errors abound. The claim is further grounded on the fact that self-sensing concrete requires replacing key sections of the concrete structure with smart materials.

#### 3.2.9. Sol-Gel Sensors

Apart from the standard classification of sensors (self-sensing concrete, fiber Bragg grating sensors, wireless and piezoelectric sensors, ultrasonic, fiber optic, and electrochemical sensors), other unconventional sensors have been proven useful in structural health monitoring. One of the notable types of unconventional sensors includes sol-gel sensors. Similar to other electrochemical sensors, sol gel-based sensors measure the concentration of chloride ions that are responsible for inducing corrosion [109]. However, the sol-gel material exhibits superior performance compared to traditional materials, and this might translate to better accuracy and reliability of the measurement values. The unique material characteristics are linked to the phase transformations from a liquid colloidal substance "sol" to a solid gel. Beyond the unique material behavior, sol-gels are ideal sensors given that they are stable, chemically inert, amorphous, and porous [109]; these properties facilitate the adsorption and retention of NaCl. However, there is no evidence of widespread utilization of sol-gels in structural health monitoring. The limited data on the application of the materials could be attributed to two factors. One, the materials might exhibit inconsistent performance as a result of the different fabrication conditions [110]. If the fabrication process is not optimized, the performance of the material would be compromised. Two, the performance of the material is inferior to other materials with a broad range of applications.

Yen et al. [109] noted that corrosion sensors coated with the sol-gel exhibited superior performance relative to traditional materials. However, the superior performance was dependent on a wide array of factors, including the nature of the precursors. Both indium chloride hydrate (InCl_3_⋅xH_2_O) and tin (IV) chloride pentahydrate (SnCl_4_ 5H_2_O sol-gel) were utilized in the former experiments [109]. In contrast, other researchers employed a unique sol-gel spray technique in place of Sn and In elements, and zirconate–titanate (PZT)/PZT composite UT films, which led to the formation of the graphite/epoxy (Gr/Ep) composites [111]. Despite the utilization of different types of sol-gel precursors, it was reported that the materials exhibited superior performance in structural health assessment. Beyond the accurate assessment of the material properties, the sol gel-based sensors helped to address the limitations of existing materials by eliminating the need for couplings, and high performance in both straight and curved surfaces, and superior performance in pulse-echo modes [111]. The preliminary positive outcomes reported by Yen et al. [109] and Kobayashi et al. [111] show that the application of sol-gel materials in electrochemical sensing was appropriate. In general, even though the deployment of sensors offers unlimited prospects in structural health monitoring, key challenges should be addressed moving forward, including fault detection architecture, algorithms, situational framework recognition [112], decision support systems applications, durability, functionality, cost, and reliability of the measurements.

## 4. Conclusions

The following insights were drawn from the critical appraisal of scholarly research relating to the development of sensors for structural health monitoring. First, agricultural environments exacerbate the risk of material failure due to frequent loading/unloading, exposure to agricultural chemicals, wastewater/animal sewer and waste, and moisture. The nature of the risks has informed the selection of the type of sensor for structural health monitoring. For example, sensors based on ultrasonic pulse velocity technology are useful and reliable in monitoring a wide range of damages, including concrete spalling, cracking, corrosion, de-bonding, delamination, and general material degradation. Second, the functionality and the reliability of sensors are contingent on the environmental variables, on-board electronics, placement of the sensors near the localized damaged areas, and the technology. The interplay of the different factors introduced fundamental constraints. For example, wireless sensors offer unique benefits, including the possibility of affordable long-term monitoring, accuracy, functionality, low maintenance, and operational costs. However, existing technologies are limited.

There are serious drawbacks, including the potential breakdown of communication between the central devices and the peripheral sensors, power interruptions, wireless sensor networks, poor fault tolerance, and data flooding in the wireless sensor network. The technical constraints are not unique to wireless sensors. ECT sensors’ application is impaired by the scarcity of suitable image reconstruction techniques, poor interpretation of the images, poor visualizations, and less detailed sensitivity maps. The challenges deprive engineers of critical information, which might, in turn, translate to inaccurate assessment of the structural health of the concrete structure. Such concerns limit ECT technology’s utility because damage can be localized to areas that are significantly exposed to bulk grain pressure, such as the silo hoppers. Similarly, the employment of electrochemical sensors has critical disadvantages—sophisticated data analysis techniques such as the Hilbert transformation of ultrasonic waves, time delays, and first arrival methods are applied to interpret the raw data. Considering that each sensor technique has its benefits and drawbacks, the selection process should be guided by the functionality, accuracy, and reliability of the data. Presently, both wireless and ultrasonic sensors satisfy the basic criteria and benchmarks; it is anticipated that future advances in technology would resolve the technical constraints in other sensor types.

## Figures and Tables

**Table 1:** Comparative analysis of the costs of different sensors [35].

Sensor Type	Sensing Principle	Response Time	Reversibility	Sensitivity	Cost
FBG based sensor	Wavelength shift	65 min	Non-reversible	Water bath	High (FBG, OSA, broadband light source)
Single-mode optical fiber	Shock waves propagation	44 h	Non-reversible	Water bath	High (pin photodetector, amplifier, spring-loaded gun)
Single-mode fiber	Propagation velocity of shock waves	5 km/s	Non-reversible	Water bath	High (Mach–Zehnder interferometer)
Polymer coated on FBG	Wavelength shift	240 min	Non-reversible	Water bath	High (FBG, OSA, broadband light source)
Polymer coated on FBG	Wavelength shift	170 min	Non-reversible	Water bath	High (FBG, OSA, broadband light source)
Multimode plastic-clad silica fiber	Gravimetric sorptivity	6.7 h	Non-reversible	Submerged in water for five days	Long fabrication time (~5 weeks)
Bare single-mode fiber	EF-FLRD ring-down technique	5 min	Reversible	10–80 mL of water	Low (single-mode fiber, photodiode detector)

**Table 2:** Frequency band, range, and cost of various types of wireless sensors [37].

Freq. Band [MHz]	Communication	Maximum	Point-to-Pont or Point-to-Multi-Point	Cost
		Communication		
	Standard	Distance		
433	Not known	Approximately 200 ft	Point-to- multi-point	Transmitters: $300 Receiver: $600
900	Serial (FHSS)	2500 ft Open field	Point-to- multi-point	Transmitter with air temperature sensor: $100 Repeater: $375 Receiver: $450
900	Serial (DSSS)	15 miles of sight line	Point-to-point and point-to-multi-point	Transmitter: $1428 Point-to-point bridge: $995 Point-to-multi-point: $1995
900	Serial	35 miles line of sight	Point-to-multi-point	Transmitter: $1775 Receiver: $1775
2400 2400	Serial Bluetooth	150 ft sight line of 30 ft to 320 ft	Point-to-point Point-to-multi-point	Transmitter: $800 Receiver: $800 Bluetooth enable wireless monitoring unit: $1795 PCMCIA Bluetooth radio card: $395

**Table 3:** Cost of corrosion as a percentage of the GDP in agriculture versus industry and service sectors [57].

Economic Regions	Agriculture CoC USD Billion	Industry CoC USD Billion	Services CoC USD Billion	Total CoC USD Billion	Total GDP USD Billion	CoC % GDP
United States	2.0	303.2	146.0	451.3	16,720	2.7%
India	17.7	20.3	32.3	70.3	1670	4.2%
European Region	3.5	401	297	701.5	18,331	3.8%
Arab World	13.3	34.2	92.6	140.1	2789	5.0%
China	56.2	192.5	146.2	394.9	9330	4.2%
Russia	5.4	37.2	41.9	84.5	2113	4.0%
Japan	0.6	45.9	5.1	51.6	5002	1.0%
Four Asian Tigers + Macau	1.5	29.9	27.3	58.6	2302	2.5%
Rest of the World	52.4	382.5	117.6	552.5	16,057	3.4%
Global	152.7	1446.7	906.0	2505.4	74,314	3.4%

**Table 4:** Presence of different OH groups in timber compounds [50].

Wood Polymer	Formula Unit	Molecular Mass	OH-Groups	OH Concentration
		(g/mol)		(mmol/g)
Cellulose	C_12_O_10_H_2_0	324	6f	18.5
Xylan	C_33_O_24_H_52_	833	12	14.4
Glucomannan	C_30_O_24_H_44_	795–1049	9–15	8.6–18.8
Lignin	C_160_O_58_H_178_	3029	24	7.9
Lignin	C_278_O_96_H_300_	5177	39	7.5
Lignin	C_301_O_110_H_335_	5713	45	7.9

**Table 5:** Risk of corrosion based on exposure to dairy manure [5].

Metal Type	Original Weight in g/cm^2^	The Average Rate of Metal Loss (g/cm^2^/month)	% of Metal Remaining (mass)	Duration of Tests (months)
Manure collection pit				
BS	1.0408	0.0474	0	22
ECS	1.0462	0.0299	0	35
BG	0.63	0	100	49
GF-600-3283		0.0094	0	35
GF-90	0.3271	0.009	0	35
SS	0.6251	0	100	49
DQ	0.4272	0.0292	0	29
Housing area				
BS	1.0408	0.0033	84.6	49
ECS	1.0462	0	100	49
BG	2.4963	0	100	49
GF-60	0.3283	0	100	49
GF-90	0.3271	0	100	49
SS	0.6251	0	100	49
DQ	0.4272	0	100	49

**Table 6:** Relationship between the parameters of interest and the techniques for monitoring the structural health of building structures [79].

Monitoring Technique		Parameters of Interest					
		Moisture Content	Decay	Insect Damage	Delaminations and Cracks	Deformations and Displacements	Strains
Electrical resistance method		x	x				
Dielectric methods		x	x				
Hygrometric method		x	x				
Wave- and vibration-based	Ultrasonic methods			x	x		
	Acoustic emissions			x	x		
	Vibration-based methods				x		
Optic methods	Digital image correlation (DIC)				x	x	x
	Signalized target measurement					x	
	Laser-based systems					x	
	Other			x			
Fibre-optic based methods				x		x	
Resistive strain sensors						x	
Self-healing materials				x			

## Data Availability

No data available.
